# *In Vivo* CAR-T Therapy for Cancer Treatment: Mechanisms, Technological Advances, and Clinical Translation

**DOI:** 10.32604/or.2026.076420

**Published:** 2026-04-22

**Authors:** Lu Hao, Jiao Lu, Jisu Xue

**Affiliations:** 1 Department of Science and Education, Shenzhen Baoan Shiyan People’s Hospital, Shenzhen, China; 2 Department of Endocrinology, Shenzhen Baoan Shiyan People’s Hospital, Shenzhen, China

**Keywords:** Chimeric antigen receptor-T, cell therapy, *in vivo* chimeric antigen receptor-T, cancer treatment, gene therapy

## Abstract

*In vivo* Chimeric Antigen Receptor (CAR)-T cell therapy reprograms a patient’s own T cells directly inside the body, bypassing the complex and costly traditional manufacturing process. This is achieved by systemically delivering viral or non-viral vectors that genetically modify endogenous T lymphocytes to produce functional CAR-T cells *de novo*. By eliminating *ex vivo* cell processing, this strategy can simplify workflows, reduce costs, improve accessibility, and allow faster treatment. Key delivery platforms include engineered lentiviral and adeno-associated viral (AAV) vectors for lasting CAR expression and targeted lipid nanoparticles (LNPs) for transient mRNA delivery. Emerging technologies like biomaterial scaffolds and ultrasound stimulation further enable localized and spatiotemporally controlled T cell engineering. Clinically, early trials in relapsed/refractory multiple myeloma and B-cell malignancies have shown strong antitumor responses, even without preconditioning chemotherapy. Remaining challenges comprise achieving precise T cell targeting, overcoming the immunosuppressive tumor microenvironment, preventing antigen escape, and managing safety risks such as vector genotoxicity or LNP reactogenicity. Future translation will depend on combining synergistic regimens, refining vector design, and implementing tunable safety controls. The aim of the study is to highlight how *in vivo* CAR-T therapy is evolving from concept to clinical reality, poised to redefine adoptive cell therapy as a scalable and widely applicable pharmacologic intervention.

## 1 Introduction

The advent of chimeric antigen receptor (CAR)-T cell therapy represents a transformative breakthrough in oncology, embodying the promise of precision immuno-oncology [1–3]. Since the first definitive clinical successes were reported, this modality has redefined treatment paradigms for patients with relapsed and refractory hematological malignancies [4,5]. To date, multiple autologous CAR-T cell products, engineered via retroviral or lentiviral vectors to target cluster of differentiation 19 (CD19) or B cell maturation antigen (BCMA), have been approved for the treatment of several B cell malignancies, including B cell acute lymphoblastic leukemia, non-Hodgkin lymphoma, and multiple myeloma [6,7]. Additionally, numerous CAR-T cell trials are in various stages of development, with over 1000 clinical trials currently ongoing worldwide [8]. The fundamental mechanism entails the genetic reprogramming of a patient’s autologous T lymphocytes to recognize and eliminate tumor cells via a single-chain variable fragment (scFv) that binds a specific surface antigen, independent of major histocompatibility complex (MHC) presentation [9]. This engineering strategy generates a “living drug” capable of robust activation, potent cytotoxicity, and long-term persistence upon encountering its target antigen [10]. Despite this progress, the expansion of autologous or allogeneic CAR-T cell therapies into broader indications and patient populations has progressed more slowly than anticipated [11]. Multiple hurdles have been identified, including the complexity of manufacturing and logistics, limited production capacity, and the requirement for chemotherapy-based lymphodepletion conditioning, all of which restrict access and limit applicability [12]. The field thus stands at another inflection point, necessitating innovations in delivery and engineering technologies to overcome these limitations and fully realize the potential of CAR-T cell therapy, and immunotherapy more broadly [13]. The complex, multi-week manufacturing process is not only extraordinarily costly and logistically cumbersome but also imposes a critical treatment delay for patients with aggressive disease [14]. Furthermore, *ex vivo* expansion can induce T cell exhaustion, compromising the fitness of the final cell product [15]. Most notably, successful application to solid tumors has proven elusive, hampered by challenges including the immunosuppressive tumor microenvironment (TME), a paucity of truly tumor-specific antigens, and difficulties in T cell trafficking and infiltration [16]. Collectively, these hurdles, spanning manufacturing, accessibility, cost, and efficacy in solid tumors, underscore an urgent clinical need for the next evolutionary leap in CAR-T and other adoptive cell transfer technologies [17,18].

*In vivo* CAR-T cell therapy is rapidly emerging as a transformative and scalable alternative to conventional approaches, obviating the need for both *ex vivo* cell manipulation and preconditioning with lymphodepleting chemotherapy [19]. The foundational principle of this strategy involves the direct *in situ* genetic engineering of a patient’s endogenous immune cells using off-the-shelf viral or non-viral vectors, thereby generating functional CAR-T cells within the body [20]. Pioneering preclinical proof-of-concept for this paradigm was established in 2017 by the group of Matthias Stephan [21], demonstrating the feasibility of *in situ* programming of leukemia-specific T cells using synthetic DNA nanocarriers. This was followed in 2018 by Christian Buchholz’s team, which reported the successful *in situ* programming of CAR-T cells using engineered lentiviral vectors, thereby validating and expanding the potential of the platform [22]. The core innovation of *in vivo* CAR-T therapy lies in its ability to bypass the entire complex logistics chain of apheresis, external cell manufacturing, and the associated lymphodepleting chemotherapy that precedes conventional CAR-T cell infusion [23]. The mechanistic underpinning of this approach relies on the systemic or local administration of advanced delivery vectors, such as lentiviral vectors, adenovirus derivatives, adeno-associated viruses (AAVs), or non-viral lipid nanoparticles (LNPs), that are engineered to traffic to lymphoid tissues or circulate systemically [24,25]. These vectors are designed to deliver their genetic payload, encoding the CAR construct, to endogenous T cells *in situ* [26]. Following cellular uptake, the payload is expressed, leading to the *de novo* synthesis and surface display of functional CARs [27]. This process enables the rapid generation of CAR-modified immune cells, which can then engage with their cognate antigen either locally within the same microenvironment where transduction occurred or, following systemic migration, at distant tumor sites [28]. This direct *in vivo* engineering paradigm confers several transformative advantages over its *ex vivo* counterpart. It dramatically streamlines the therapeutic pipeline, compressing the timeline from diagnosis to treatment initiation from several weeks to a matter of days [29]. This expedited process, coupled with the elimination of costly, labor-intensive, and facility-dependent Good Manufacturing Practice (GMP) cell culture, is projected to substantially reduce the overall economic burden of therapy, thereby enhancing its global accessibility and scalability. Furthermore, by circumventing the *ex vivo* expansion phase, a process known to promote T cell differentiation and exhaustion, *in vivo*-generated CAR-T products may be derived from a more naïve and stem-like T cell repertoire, potentially yielding a population with superior *in vivo* persistence and sustained effector function [30]. Finally, the ability to administer repeated doses transforms CAR-T therapy from a one-time cellular product into a manageable pharmacotherapy, opening the door to chronic treatment regimens and dose titration, which is largely infeasible with current *ex vivo* products [31].

In light of the remarkable and rapid progression of this field, this review aims to deliver a comprehensive and critical perspectives of *in vivo* CAR-T cell therapy in cancer research, charting its journey from fundamental mechanistic insights to the forefront of clinical translation. We have structured our scope to bridge disciplinary divides, offering a resource that is equally pertinent to basic immunologists, translational scientists, clinical oncologists, and bioengineers. Our review will commence with a systematic dissection and comparative evaluation of the challenges of *ex vivo* CAR-T cell therapy and the rational and technological platforms that enable *in vivo* T cell programming. We then discussed the latest viral and non-viral strategies, particularly LNPs for mRNA and DNA delivery, as well as other innovative modalities such as implantable biomaterial scaffolds and ultrasound-mediated transfection technologies. Subsequently, we critically synthesized the burgeoning body of emerging clinical trial evidence. This synthesis will place a dedicated focus on the evolving safety profiles, including the unique manifestations and management of cytokine release syndrome and the nuanced risks of on-target, off-tumor toxicity, alongside efficacy outcomes across a spectrum of hematological malignancies and solid tumor models. Finally, the review will confront the pivotal biological and technical challenges that currently gatekeep widespread clinical adoption. We will delve into the paramount hurdle of achieving stringent *in vivo* T cell selectivity to mitigate off-target transduction, explore emerging strategies for controlling the expansion and long-term persistence of engineered cells, interrogate the ongoing quest for ideal tumor antigens in the context of solid malignancies, and navigate the complex, evolving regulatory landscape for these novel therapeutic agents. By integrating these multifaceted perspectives, this article seeks not only to delineate the current state-of-the-art but also to chart a clear and informed pathway for the future trajectory of *in vivo* CAR-T therapies. Our ultimate goal is to illuminate how this paradigm can be refined to become a more scalable, affordable, and potent therapeutic modality across the entire spectrum of human cancers.

To ensure transparency in the literature identification process, this review was conducted based on a comprehensive search of peer-reviewed articles and clinical trials published primarily between 2020 and ending of 2025. Relevant publications were retrieved from major scientific databases, including PubMed, Web of Science, and Google Scholar, using key search terms such as “*in vivo* CAR-T therapy”, “cancer immunotherapy”, “clinical translation”, “viral vectors”, “lipid nanoparticles”, and “clinical trials”. The search strategy emphasized recent studies, landmark trials, and technological advances reported up to the ending of 2025. Inclusion criteria focused on original research and clinical investigations addressing the mechanisms, delivery platforms, clinical outcomes, and challenges of *in vivo* CAR-T therapy. Articles that were not directly relevant to *in vivo* CAR-T approaches, published in languages other than English, or lacking sufficient methodological detail were excluded. As a narrative review, this work synthesizes current evidence and emerging trends rather than adhering to a formal systematic review protocol, with the aim of providing an integrated and forward-looking perspective on the field.

## 2 Limitations of Conventional *Ex Vivo* CAR-T Cell Therapy

**Figure 1:**
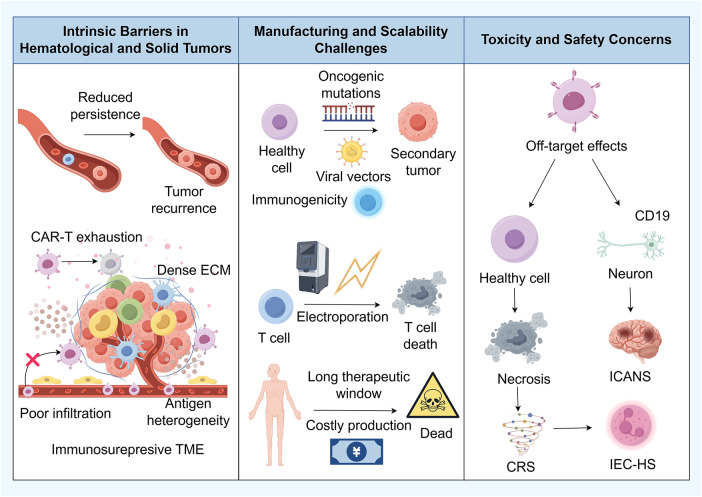
Limitations of conventional *ex vivo* CAR-T cell therapy in cancer treatment. Major challenges associated with CAR-T cell therapy comprised intrinsic barriers in hematological and solid tumors such as CAR-T exhaustion, reduced persistence, tumor recurrence, dense ECM, antigen heterogeneity, and an immunosuppressive TME. It further outlines manufacturing and scalability challenges, including oncogenic mutations, risks to healthy cells, potential secondary tumors, immunogenicity, electroporation effects on T cells, a long therapeutic window, and costly production. Finally, the diagram addresses toxicity and safety concerns, covering off-target effects, impacts on neurons and healthy cells, necrosis, and specific adverse events such as ICANS, CRS, and IEC-HS. Abb: CAR-T: Chimeric Antigen Receptor T-cell; ECM: Extracellular Matrix; TME: Tumor Microenvironment; ICANS: Immune Effector Cell-Associated Neurotoxicity Syndrome; CRS: Cytokine Release Syndrome; IEC-HS: Immune Effector Cell-Associated Hemophagocytic Syndrome; CD: Cluster of Differentiation. This figure is created by Figdraw.

Despite the transformative success of *ex vivo* CAR-T cell therapy in treating hematological malignancies, the broader application of CAR-T cell therapy is constrained by several interconnected challenges. These include the suboptimal efficacy against solid tumors, limited CAR-T cell persistence, frequent antigen escape, complex and costly *ex vivo* manufacturing process, and serious treatment-related toxicities [32,33] (Fig. 1). Addressing these multifaceted hurdles is critical for advancing the next generation of cellular immunotherapies.

### 2.1 Intrinsic Barriers in Hematological and Solid Tumors

A significant clinical challenge in hematological cancers is the reduced persistence of *ex vivo*-expanded CAR-T cells [34]. The median duration of detectable CAR-T cells in the peripheral blood of patients is often limited to 3–6 months [35]. This transient engraftment can create a therapeutic window that allows for tumor immune escape and eventual relapse [36]. This phenomenon is starkly illustrated in diffuse large B-cell lymphoma (DLBCL), where approximately 40% of patients who initially achieve a remission following anti-CD19 CAR-T cell therapy subsequently experience disease recurrence, frequently associated with the loss of functional CAR-T cells from the periphery, a state known as “CAR-T cell exhaustion [37]”. This exhaustion is characterized by an upregulation of inhibitory receptors (e.g., PD-1, TIM-3, LAG-3) and a progressive loss of effector functions, including cytokine production and cytotoxic capacity [38].

Although early-phase clinical trials have provided glimpses of efficacy in specific malignancies such as glioma, neuroblastoma, and sarcoma, the overall response rates remain modest and often unsustained [39–41]. This constrained efficacy and limited persistence of CAR-T cells in the context of solid tumors can be ascribed to a series of interconnected biological barriers inherent to the TME (Fig. 1). A primary limiting factor is the inefficient homing of systemically administered CAR-T cells to tumor sites, a process often compromised by the downregulation of homing chemokines by the tumor itself and the frequent lack of corresponding chemokine receptors on CAR-T cells, culminating in inadequate tumor-specific trafficking and infiltration [42]. Upon nearing the tumor mass, CAR-T cells encounter formidable physical obstacles, including a dense stromal matrix and aberrant, dysfunctional vasculature, which collectively impede their extravasation and penetration into the tumor core, thereby sequestering them from their cellular targets [43]. Furthermore, the established TME is intrinsically immunosuppressive and metabolically hostile [11]. Defined by conditions such as hypoxia, acidity, and nutrient depletion, along with an abundance of inhibitory factors like transforming growth factor-β (TGF-β), this milieu actively recruits and sustains immunosuppressive cell populations, including regulatory T cells (Tregs) and myeloid-derived suppressor cells (MDSCs) [44,45]. This constellation of adverse conditions synergistically undermines CAR-T cell proliferation, effector functions, and survival, ultimately precipitating a state of functional exhaustion or anergy. Compounding these challenges is the pervasive heterogeneity of antigen expression characteristic of solid tumors, which readily facilitates immune escape. The loss or downregulation of the target antigen in a subset of tumor cells enables the outgrowth of antigen-negative clones that remain invisible to monospecific CAR-T cells [46–49].

### 2.2 Manufacturing and Scalability Challenges

The clinical manufacturing of autologous CAR-T cells involves a laborious, multi-step *ex vivo* process that encompasses leukapheresis, T-cell activation, genetic modification for CAR expression, and extensive cellular expansion [50]. This intricate procedure necessitates sophisticated clinical-grade infrastructure, rigorous quality control protocols, and substantial financial investment, collectively contributing to the high treatment costs and limited scalability that currently characterize this therapeutic modality [51]. A pivotal stage in this manufacturing pipeline is the genetic engineering of T cells, achieved primarily through viral or non-viral transduction methods [52]. Viral vectors, particularly gamma-retroviruses and lentiviruses, remain the most widely utilized platforms owing to their high transduction efficiency and capacity for stable genomic integration, which facilitates durable CAR expression [53]. However, their clinical translation is impeded by several inherent limitations, including immunogenicity, risks of insertional oncogenesis, complex and costly production workflows, and constrained cargo capacity for larger genetic payloads [54,55]. In response to these challenges, significant efforts have been directed toward developing non-viral gene delivery platforms [56]. Techniques such as electroporation present a safer and more flexible alternative, unconstrained by the size limitations typical of viral vectors [57]. By applying controlled electrical pulses to create transient pores in the cell membrane, electroporation enables efficient intracellular delivery of nucleic acid cargoes, including plasmid DNA or mRNA [58,59]. This approach offers substantial cargo capacity and is particularly well-suited for rapid, transient CAR expression via mRNA delivery [60]. Nevertheless, its application is counterbalanced by considerable cytotoxicity; the required electrical field parameters can induce significant membrane disruption and cytoplasmic leakage, adversely affecting cell viability, altering gene expression profiles, and potentially compromising the functional fitness of the final CAR-T product [61–63] (Fig. 1).

### 2.3 Toxicity and Safety Concerns

A fundamental safety concern is the limited spatial and temporal control over CAR-T cell activity following infusion, which may lead to uncontrolled inflammatory responses and collateral damage to healthy tissues. The most prominent adverse events include cytokine release syndrome (CRS), immune effector cell-associated neurotoxicity syndrome (ICANS), and immune effector cell-associated hemophagocytic lymphohistiocytosis-like syndrome (IEC-HS) [64,65]. CRS is characterized by a massive elevation of pro-inflammatory cytokines, with interleukin-6 (IL-6) serving as a key mediator [66]. Notably, the cytokine storm is not solely attributable to direct secretion by activated CAR-T cells. Emerging evidence underscores the critical role of pyroptosis, a gasdermin-mediated, pro-inflammatory form of cell death, during CAR-T-mediated tumor killing [67]. Pyroptosis in target cells results in the release of damage-associated molecular patterns (DAMPs), which hyperactivate antigen-presenting cells (APCs) and further amplify the secretion of cytokines such as IL-1β and IL-6, thereby exacerbating the CRS cascade [68]. The correlation between gasdermin E expression levels and CRS severity highlights the central contribution of pyroptosis to its pathogenesis [69]. Clinically, CRS presents with high fever, hypotension, hypoxia, and may progress to multi-organ failure [70]. Standard management involves anti-IL-6 receptor antagonists (e.g., tocilizumab) and corticosteroids [71]. Promisingly, pre-emptive strategies such as pretreatment with the anti-CD19 monoclonal antibody tafasitamab have demonstrated efficacy in mitigating CRS severity in CD19-directed CAR-T therapy by modulating antigen exposure, offering a novel means to decouple anti-tumor potency from inflammatory toxicity [72]. ICANS is frequently associated with CAR-T cells targeting antigens such as CD19 or CD22, which may be expressed on neural tissues or provoke endothelial activation and blood-brain barrier disruption. It manifests as a spectrum of neurological symptoms, ranging from encephalopathy and aphasia to cerebral oedema [73]. Beyond supportive measures and corticosteroids, emerging interventions such as IL-18 blockade and surface PEGylation of CAR-T cells to modulate their activation profile have shown therapeutic potential [74–76]. IEC-HS, often coexisting with severe CRS, is defined by fulminant macrophage activation and a hyperinflammatory state, and may be managed with corticosteroids and the IL-1 receptor antagonist anakinra [77]. Additional safety considerations include the risk of graft-versus-host disease in allogeneic settings, on-target/off-tumor toxicity, anaphylactic reactions to murine-derived scFv domains, theoretical concerns regarding viral insertional oncogenesis, and the current inadequacy of real-time monitoring methodologies [78] (Fig. 1). A concerted research effort is therefore essential to advance the development of smarter, safer, and more precisely controllable CAR-T platforms capable of mitigating these multifaceted risks [79].

## 3 The Principle and Advancement of *In Vivo* CAR-T Therapy

Due to the limitations of current *ex vivo* CAR T-cell therapies, there is a growing urgency to develop novel strategies that address the suboptimal efficiency, complexities of *ex vivo* manufacturing processes and mitigate associated systemic toxicities. One particularly promising direction involves the *in vivo* generation of CAR T cells, which, despite remaining technically challenging, represents a potentially transformative alternative. This approach could significantly streamline and standardize manufacturing, potentially enabling CAR-T therapy to become a universally applicable “off-the-shelf” treatment. Furthermore, *in situ* reprogramming of CAR T cells may circumvent the functional exhaustion often observed with conventional methods, where repeated *in vitro* expansion and activation prior to reinfusion impair T-cell fitness [80]. By contrast, CAR T cells generated directly *in vivo* are neither extensively manipulated nor subjected to abrupt activation, expanding instead through more physiologically gradual kinetics, which could better preserve their effector functions and longevity.

### 3.1 Basic Principle and Mechanism of In Vivo CAR-T Therapy

The fundamental objective of *in vivo* CAR-T therapy is to genetically reprogram a patient’s endogenous T lymphocytes to express CARs directly within the body, thereby circumventing the need for cell extraction [81]. This process can be conceptualized as a multi-stage biological cascade comprising targeted delivery, cellular transduction, and functional engraftment coupled with expansion [30]. It begins with the administration of a genetically engineered vector carrying the CAR transgene, which serves as the cornerstone of the approach. This vector must be meticulously designed to navigate the circulatory system, home to lymphoid tissues where T cells predominantly reside, and efficiently enter target T cells. The CAR transgene may be delivered in the form of DNA, typically packaged within viral vectors or non-viral nanoparticles, or as messenger RNA (mRNA), most commonly encapsulated in LNPs [82]. The choice between DNA and mRNA profoundly influences the kinetics and persistence of CAR expression. DNA-based systems, especially those employing integrating viral vectors such as lentiviruses, enable stable genomic integration, facilitating long-term CAR expression and the potential establishment of a self-renewing CAR-T cell population [83]. In contrast, mRNA-based delivery leads to transient but high-level CAR expression from episomal transcripts in the cytoplasm, typically lasting from several days to a few weeks. While this transient expression may constrain long-term efficacy, it provides an inherent safety mechanism by limiting the risk of persistent on-target/off-tumor activity or uncontrolled T-cell expansion [84]. A critical challenge lies in achieving selective transduction of T cells following systemic or localized vector administration [85]. Advanced targeting strategies are essential to this end. Viral vectors may be pseudotyped with engineered envelope proteins that bind receptors enriched on T cells, whereas non-viral LNPs can be functionalized with targeting ligands such as antibodies or antibody fragments directed against T-cell surface markers like CD3 or CD8 [86]. Such directed targeting is vital to minimize off-target transduction in other immune or parenchymal cells, which could lead to unintended clearance of the vector by APCs or neutralization by pre-existing antibodies [87]. Upon successful payload delivery into the T-cell cytoplasm, cellular machinery directs the subsequent steps [88]. For DNA vectors, the CAR cassette must traverse into the nucleus to be transcribed into mRNA and then translated into protein [89]. In the case of mRNA-LNPs, the mRNA bypasses nuclear entry and is directly translated by ribosomes [31]. The newly synthesized CAR protein undergoes folding, glycosylation, and transport to the T-cell surface, where it integrates into the plasma membrane as a functional receptor [90]. The resulting CAR-T cell population then becomes activated and undergoes clonal expansion *in vivo*, driven by engagement with tumor-associated antigens. This physiological activation within the native immune milieu is hypothesized to support a less differentiated, stem-cell-like phenotype, which may endow *in vivo*-generated CAR-T cells with enhanced persistence and functional resilience compared to their *ex vivo* counterparts, which often exhibit terminal differentiation after extensive artificial expansion [91].

### 3.2 Comparative Overview: Ex Vivo vs. In Vivo CAR-T

**Figure 2:**
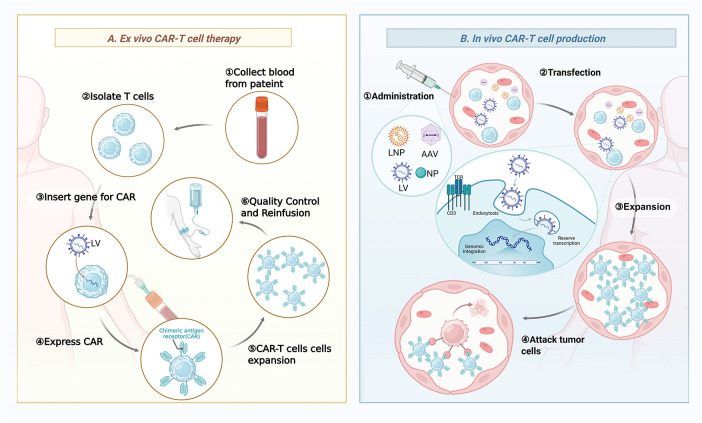
Schematic comparison of *ex vivo* and *in vivo* approaches for generating CAR-T cells. (**A**) *Ex vivo* CAR-T cell therapy involves collecting patient blood, isolating T cells, and genetically modifying them outside the body to express CARs using LV, followed by cell expansion, quality control, and reinfusion into the patient. (**B**) *In vivo* CAR-T cell production is achieved through direct administration of delivery systems such as LNP or LVP, facilitating transfection, pioneer transcription, and expansion of CAR-T cells inside the body, ultimately enabling them to attack tumor cells. Abb: LV, lentiviral vector; LNP, lipid nanoparticle; LVP, lentiviral particle. This figure is created by Biorender.

The distinction between *ex vivo* and *in vivo* CAR-T manufacturing transcends the mere site of cellular modification, reflecting instead a foundational divergence in therapeutic philosophy, with profound consequences for scalability, cost, product characteristics, and clinical applicability (Table 1, Fig. 2).

**Table 1:** Comparison of *ex vivo* CAR-T and *in vivo* CAR-T.

Features	*Ex vivo* CAR-T therapy	*In vivo* CAR-T therapy
Manufacturing process	Multi-step, centralized, patient-specific (autologous). Complex logistics from apheresis to infusion.	Single-step, *in situ* engineering. Administered as an off-the-shelf formulation.
Production timeline	Protracted (2–4 weeks). Creates a critical treatment delay.	Condensed (theoretical timeline of days). Enables rapid intervention.
Cost & Infrastructure	Extremely high cost; requires GMP facilities and specialized labor.	Potentially significantly lower cost; leverages scalable pharmaceutical manufacturing.
T cell product	Homogeneous, defined cell product. Quality and dose are controlled pre-infusion.	Heterogeneous, polyclonal. Dose and phenotype are influenced by host factors.
T cell phenotype	Often skewed towards effector-memory and terminally differentiated phenotypes due to *ex vivo* culture.	Hypothesized to include more naïve and stem-cell memory T cells, potentially enhancing persistence.
Safety & control	Defined cell dose; toxicities manageable but can be severe (CRS, ICANS). Limited control post-infusion.	Less predictable pharmacokinetics/pharmacodynamics. Potential for off-target transduction. Offers potential for re-dosing and titratable, transient activity (e.g., with mRNA).
Accessibility	Limited to major academic centers in high-income countries.	Potential for global accessibility, similar to conventional biologics.

Note: Abb: CAR: Chimeric Antigen Receptor; CRS: Cytokine Release Syndrome; GMP: Good Manufacturing Practice; ICANS: Immune effector Cell-Associated Neurotoxicity Syndrome.

A primary advantage of the *in vivo* platform lies in its streamlined manufacturing and logistical profile. By circumventing the need for leukapheresis, cell shipment, and extended *ex vivo* culture, this approach obviates the costly and complex infrastructure that currently constrains widespread adoption of *ex vivo* CAR-T therapies [92]. This shift from a patient-specific cellular product to a standardized, off-the-shelf injectable holds considerable potential to markedly reduce treatment expenses and broaden global access, possibly enabling administration even in community oncology settings. Biologically, the *in vivo* strategy may also yield a qualitatively distinct T-cell product [93]. *Ex vivo* manufacturing typically involves repeated stimulation that drives T cells toward a more differentiated and exhausted state, potentially undermining their long-term persistence and functional durability in patients. In contrast, *in situ* engineering allows T cells to be activated and expand within their native immunological microenvironment, a process thought to better maintain a reservoir of less-differentiated, long-lived memory T cells, which may in turn support more sustained clinical responses [94]. Furthermore, the capacity for redosing, particularly with non-integrating, transient systems such as mRNA-loaded LNPs, introduces an unprecedented degree of therapeutic control [93]. Clinicians could potentially titrate dosing in response to clinical dynamics, mitigate toxicity through dose interruption, or implement chronic treatment regimens resembling conventional pharmacotherapy, a level of flexibility absent from the typically single-administration framework of most existing *ex vivo* products [95].

Nevertheless, these substantial advantages are tempered by several unique challenges inherent to the *in vivo* paradigm. The foremost obstacle remains the achievement of efficient and selective T-cell transduction within a complex physiological environment. Pre-existing immunity to viral vectors may lead to neutralization before target engagement, while nonspecific clearance by hepatic Kupffer cells or splenic macrophages can sequester and degrade nanoparticle-based vectors, substantially diminishing transduction efficiency. The risk of off-target transduction in non-T cells, such as B cells or myeloid lineages, also presents a serious safety consideration, necessitating meticulous vector design to enhance specificity. Moreover, the pharmacokinetics and pharmacodynamics of *in vivo*-generated CAR-T cells are inherently less predictable [96]. The resultant functional CAR-T cell dose is influenced by interpatient variables including endogenous T-cell counts, overall immune status, and the presence of neutralizing antibodies, a degree of variability that stands in stark contrast to the precisely defined cell doses administered in *ex vivo* therapies [97]. Practical strategies to circumvent this include capsid serotype switching to less prevalent variants (e.g., AAV-LK03, AAVrh74) with lower pre-existing immunity in human populations, as explored in clinical gene therapy trials for hemophilia and muscular dystrophy [98]. In selected cases, transient immunomodulation with corticosteroids or more intensive immunosuppressive regimens (e.g., mycophenolate mofetil, tacrolimus) during vector administration can be employed to blunt adaptive immune responses against the capsid and transgene, a strategy supported by lessons from *in vivo* gene therapy for inherited retinal diseases [99,100].

## 4 Key Technological Platforms for *In Vivo* CAR-T Engineering

**Figure 3:**
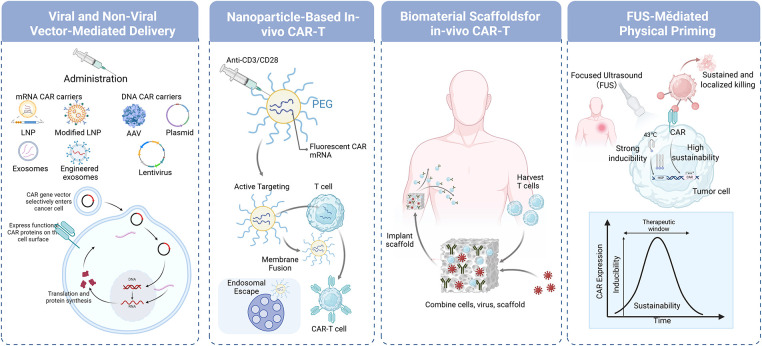
Strategies for *in vivo* generation of CAR-T cells using various delivery platforms. The figure outlines various vector-mediated approaches, including LNP, engineered exosomes, AAV, plasmids, and lentivirus, to deliver CAR-encoding mRNA or DNA into T cells *in vivo*. These systems facilitate processes such as membrane fusion, endosomal escape, and intracellular CAR protein expression. Additional strategies include nanoparticle-based CAR-T production, biomaterial scaffolds for localized and sustained CAR-T activity, and FUS-mediated physical priming to enhance CAR expression and tumor cell targeting. Abb: LNP, lipid nanoparticle; AAV, adeno-associated virus; PEG, polyethylene glycol; FUS, focused ultrasound. This figure is created by Biorender.

To overcome the limitations of conventional *ex vivo* CAR-T cell therapies, a novel strategy for generating CAR-T cells directly *in vivo* has been developed (Fig. 2). Unlike traditional *ex vivo* methods, this innovative technology employs targeted delivery systems, such as engineered viral vectors and nanoparticles, to selectively introduce CAR genes into a patient’s T cells inside the body. *In vivo* CAR-T cell therapy has demonstrated promising antitumor efficacy and a favorable safety profile in animal studies. For example, in a mouse model of acute lymphoblastic leukemia, systemic injection of an adeno-associated virus (AAV) vector carrying the CAR gene led to successful *in vivo* generation of functional CAR-T cells, which effectively eliminated leukemia cells without inducing significant systemic toxicity [101]. The successful implementation of *in vivo* T cell engineering relies on gene delivery systems that meet specific criteria, including accurate T cell targeting, efficient gene transfer, and minimal toxicity. To date, a variety of targeted delivery platforms, primarily based on viral vectors or nanocarriers, have been developed for in situ generation of CAR-T cells (Fig. 3 and Table 2).

**Table 2:** Comparison of key delivery platforms for *in vivo* CAR-T engineering.

Platform	Targeting Strategy	Persistence	Safety Concerns	Translational Status	Refs
Lentivirus	Engineered envelope proteins (e.g., NiV glycoprotein with CD8-specific DARPin/scFv)	Integrating (Long-term)	Insertional mutagenesis, immunogenicity, off-target transduction	Early clinical (e.g., INT2104, ESO-T01)	[102–104]
AAV	Capsid engineering for tropism modification	Episomal (Long-term expression)	Pre-existing immunity, off-target effects, immunogenicity	Early clinical & Preclinical	[101,105,106]
LNP	Antibody-conjugated or immunotropic lipid formulations	Transient (mRNA-based)	Reactogenicity, infusion reactions, hepatotoxicity	Early clinical (e.g., CPTX2309)	[107,108]
Exosomes	Surface-displayed targeting ligands (e.g., anti-CD3/CD28 scFv)	Transient (mRNA-based)	Manufacturing scalability, payload loading efficiency, biodistribution control	Preclinical	[109,110]
Scaffolds	Localized, sustained release of CAR construct + T-cell ligands	Sustained (depends on cargo)	Biocompatibility, local inflammation, surgical implantation	Preclinical	[111–113]

Note: Abbreviations: AAV: Adeno-associated virus; DARPin: Designed ankyrin repeat protein; LNP: Lipid nanoparticle; NiV: Nipah virus; scFv: single-chain variable fragment

### 4.1 Viral Vector-Mediated Delivery

#### 4.1.1 Lentivirus

Lentiviral vectors pseudotyped with vesicular stomatitis virus glycoprotein (VSV-G) currently represent the most widely used platform for T cell engineering [114]. The broad tropism of VSV-G-pseudotyped lentiviruses stems from the ability of VSV-G to bind the low-density lipoprotein receptor, which is ubiquitously expressed on mammalian cells [114]. Notably, VSV-G mediates both receptor binding and membrane fusion through a single protein, and mutagenesis of key amino acid residues can ablate its native receptor recognition while retaining fusogenic activity [115]. This dual functionality contrasts with glycoproteins from the paramyxovirus family, such as the H and F proteins of measles virus (MV) [116] and the G and F proteins of Nipah virus (NiV) [117], where receptor binding and membrane fusion are segregated into separate subunits. This structural separation facilitates engineering of cell-type-specific entry mechanisms, making paramyxovirus glycoproteins highly promising for targeted vector design.

Engineered NiV glycoproteins have been leveraged to enhance lentiviral gene delivery activity while enabling T cell-specific transduction. By displaying CD4- or CD8-specific binding domains, NiV-pseudotyped vectors achieved over 99% specificity for CD8+ T cells [118]. The first T cell-targeted lentiviral system for *in vivo* CAR-T cell generation employed a NiV-based vector displaying a designed ankyrin repeat protein (DARPin) specific to the CD8α chain [102]. A single systemic administration of this CD8-targeted vector enabled *in vivo* generation of functional human CD19-specific CAR-T cells in humanized mice, leading to effective clearance of primary B cells and lymphoma cells from circulation and bone marrow [103]. Subsequent studies using lentiviral vectors targeted to other T cell markers have further expanded the scope of *in vivo* CAR therapy. For instance, anti-CD4-targeted lentiviral vectors mediated highly efficient tumor cell elimination in preclinical models, with activity comparable or superior to that achieved with CD8-targeted vectors [119]. Targeting CD3 using agonistic single-chain antibodies enabled both selective T cell binding and concurrent activation [120]. However, CD3 engagement typically induces rapid internalization and receptor downregulation, limiting transduction efficiency. To address this, the tyrosine kinase inhibitor dasatinib was employed during transduction to transiently suppress TCR signaling, thereby preserving CD3 surface expression and enhancing gene transfer [121].

Although most preclinical *in vivo* CAR-T studies have employed B cell-targeted CARs, recent work demonstrates the utility of this platform in T cell malignancies. In a model of angioblastic T cell lymphoma composed of CD4+ tumor cells, *in vivo* delivery of CD4-directed CARs using CD8-targeted lentiviral vectors mediated effective tumor control [104]. Importantly, restricting CAR delivery to CD8+ T cells prevented transduction of malignant CD4+ cells, thereby circumventing a potential mechanism of treatment resistance. While mouse models have been essential for validating these approaches, large animal studies, particularly in non-human primates (NHPs), have proven critical for translational optimization, informing platform design and dosing regimens in preparation for clinical testing [122].

Additional strategies continue to be explored. A lentiviral vector pseudotyped with a mutated Sindbis virus (SINV) envelope and retargeted to CD3+ T cells via a bispecific tandem Fab molecule achieved specific transduction, though with lower efficiency compared to other engineered platforms [123]. Similarly, the Cocal virus glycoprotein, a VSV-G homolog with enhanced serum inactivation resistance, has been used for vector pseudotyping [122]. However, in the absence of targeted mutations, its broad tropism may increase the risk of off-target transduction. Collectively, these advances underscore the ongoing evolution of lentiviral vector engineering toward enhanced specificity, efficiency, and translational relevance for *in vivo* CAR-T cell generation.

#### 4.1.2 Adeno-Associated Virus

Adeno-associated virus (AAV) vectors have established themselves as a leading platform for *in vivo* CAR-T cell generation, combining efficient gene delivery with a favorable safety profile. As non-enveloped, single-stranded DNA viruses with a compact genome of approximately 4.7 kb, AAVs can be genetically reprogrammed to deliver therapeutic transgenes into specific target cells [124,125]. Following transduction, AAV genomes are maintained predominantly as episomes within the host nucleus, supporting sustained transgene expression without genomic integration. A key advantage over adenoviral vectors is their reduced immunogenicity and diminished capacity to provoke inflammatory immune responses [126]. Moreover, recombinant AAV vectors are stripped of viral coding sequences involved in replication and pathogenicity and do not require synthetic lipids or chemical modifications for effective transduction, attributes that collectively underpin their excellent safety and low immunogenicity [127]. Extensive clinical evidence from gene therapy trials continues to affirm the safety and efficacy profiles of recombinant AAVs in human applications [128].

The combination of high transduction efficiency and stable transgene expression renders AAV vectors particularly suitable for *in vivo* CAR-T cell therapy [80]. A critical factor in AAV tropism is the capsid structure, which can be systematically engineered to enhance cell-type specificity. In a notable recent demonstration, a single infusion of an AAV vector encoding a CAR led to the direct *in vivo* generation of functional CAR-T cells in a humanized mouse model of T-cell leukemia, resulting in sustained tumor regression and the establishment of antitumor immunity. This strategy, termed AAV-delivered CAR gene therapy (ACG), eliminates the need for *ex vivo* cell manipulation and lymphodepletion, offering a simplified and potentially more broadly applicable platform for CAR-T therapy [101]. It should be noted, however, that this particular study did not incorporate T-cell redirection modifications, leaving off-target effects an area warranting further investigation. Complementing these efforts, two independent studies have developed Ark313, an evolved AAV variant optimized for efficient genetic engineering of murine T cells *in vivo*. Ark313 outperforms natural serotypes in transduction efficiency and supports stable transgene integration, including of large DNA donor templates, across diverse T cell subsets, even in immunocompetent contexts. By enabling CRISPR-Cas-mediated knockout and homology-directed repair, this system allows the generation of CAR-T and TCR-T cells entirely *in vivo* through gRNA-directed targeting of the TRAC locus, thereby offering a powerful tool for preclinical immunotherapy testing and T cell research [105,106]. Additionally, the engineered homing endonuclease system ARCUS nuclease represents another promising approach for targeted gene integration. With its compact architecture and regular spacer arrangement, ARCUS provides a shorter alternative to the CRISPR-Cas9 system that is more amenable to AAV packaging. Together, these advances underscore the transformative potential of engineered AAV systems in enabling efficient, scalable, and physiologically relevant *in vivo* T cell programming for both investigative and therapeutic applications.

### 4.2 Nanoparticle-Based Strategies

RNA-based platforms have emerged as a versatile strategy for *in vivo* immune cell engineering, utilizing either linear or circular mRNA formats delivered through tissue-tropic or antibody-functionalized LNPs [129]. While early proof-of-concept studies employed polymer-based nanoparticles decorated with anti-CD3 or anti-CD8 antibodies to deliver CD19-targeting CAR mRNA [130], LNPs have since become the leading delivery platform due to extensive manufacturing experience from mRNA vaccines and continued innovation in ionizable lipid design [131]. Recent advances in biodegradable ionizable lipids with improved tolerability and reduced reactogenicity now enable re-dosing regimens and support desired therapeutic profiles. Further refinement in targeting has been achieved through antibody-functionalized LNPs for precise T cell engineering, as well as immunotropic LNP variants identified via high-throughput screening, which exhibit innate tropism for specific immune cell subsets [132]. Structurally, LNPs are spherical vesicles composed of single or multiple phospholipid bilayers [133], encapsulating mRNA to protect it from degradation and maintain stability *in vivo* [134]. Common preparation methods include electroformation, hydration, and extrusion, with the thin-film hydration technique representing a classical approach wherein phospholipids and cholesterol self-assemble into bilayers upon hydration [135]. Functionally, LNPs enhance delivery efficiency through membrane fusion and facilitate cytoplasmic mRNA release via pH-sensitive endosomal escape. By surface modification with targeting ligands, LNPs can achieve cell-type-specific interactions [136], enabling transient transgene expression *in vivo*. This transient CAR expression reduces risks associated with permanent CAR-T cell activity, such as cytokine release syndrome [137].

A landmark study by Rurik et al. in 2022 demonstrated the therapeutic potential of *in vivo* CAR-T cell generation using CD5-targeted mRNA-LNPs, which produced anti-fibroblast activation protein (FAP) CAR-T cells in a mouse model of heart failure, resulting in reduced cardiac fibrosis and functional recovery [138]. However, conventional LNPs exhibit strong hepatic accumulation, with 80%–95% of intravenously administered particles accumulating in the liver, limiting their utility for non-hepatic diseases. To address this, an anti-CD3-modified LNP system carrying a plasmid encoding both CD19-CAR and IL-6 shRNA was developed, enabling *in vivo* generation of functional CAR-T cells while mitigating IL-6-mediated toxicity [139]. LNP-based CAR-T cell platforms offer distinct advantages over viral vectors, including avoidance of insertional mutagenesis, reduced immunogenicity, simplified production, and lower manufacturing costs. The recent FDA approval of an LNP-siRNA therapeutic for hereditary transthyretin amyloidosis underscores the clinical translatability of LNP platforms. Nevertheless, challenges remain in T cell transfection efficiency and endosomal escape [140,141]. Recent progress includes the development of LNPs for *in situ* engineering of myeloid cells with TROP2-targeting CARs, which trigger anti-tumor immunity in solid tumor models [107], as well as novel ionizable lipids and antibody-conjugated LNPs that enable efficient extrahepatic delivery and functional CAR-T cell generation *in vivo* [108]. Companies such as Sanofi are also advancing targeted LNP (tLNP) platforms incorporating antibody fragments against CD8^+^ T cells and structurally optimized ionizable lipids, achieving enhanced cell-specific transfection and tunable CAR expression with broad therapeutic potential [142]. These innovations collectively highlight the modularity, specificity, and translational promise of LNP systems for *in vivo* cell engineering.

Beyond lipid-based nanosystems, polymeric nanoparticles have been widely adopted for *in vivo* CAR-T cell generation owing to their structural versatility and tunable surface properties. These nanocarriers can encapsulate nucleic acids through electrostatic interactions, typically via cationic polymers such as polyethyleneimine (PEI), poly(2-dimethylaminoethyl methacrylate) (PDMAEMA), or poly(β-amino ester) (PβAE), which protect genetic cargo from nuclease degradation and facilitate intracellular release. Although polymeric nanoparticles offer advantages in loading capacity, customizability, and biocompatibility, their translational potential has been limited by suboptimal T cell transfection efficiency and specific cellular barriers. Nevertheless, in a proof-of-concept demonstration, systemic administration of PβAE nanoparticles encapsulating mRNA encoding a prostate tumor–targeted CAR significantly prolonged survival in murine models, highlighting their therapeutic potential [21].

Biofilm-coated biomimetic carrier systems represent an emerging class of delivery platforms capable of mimicking the homing behaviors of endogenous cells, such as the targeted migration of platelets and mesenchymal stem cells to injury sites, or the spleen-specific homing of red blood cells [143]. By functionalizing these carriers with T cell–specific ligands, researchers have achieved precise *in vivo* delivery of CAR constructs, enabling spatially controlled T cell engineering [144]. Exosomes, natural extracellular vesicles ranging from 40 to 160 nm in diameter, have also emerged as promising vectors for *in vivo* gene delivery [145]. Their inherent stability, low immunogenicity, and biocompatibility make them particularly suitable for delivering CAR-encoding mRNA. In a recent advance, exosomes were engineered to display anti-CD3/CD28 single-chain variable fragments (scFvs) on their surface and to encapsulate CAR mRNA via a bacteriophage MS2–LAMP-2B fusion system. These modified exosomes successfully generated functional CAR-T cells *in vivo*, demonstrating potent antitumor activity and underscoring the potential of exosomal platforms for decentralized CAR-T cell production [109]. Another group developed tumor antigen-stimulated dendritic cell-derived exosomes (tDC-Exo) displaying both anti-CD3 and anti-EGFR moieties. This platform not only activates endogenous T cells but also promotes their binding to tumor cells, mimicking key aspects of conventional CAR-T therapy while leveraging natural immune coordination mechanisms to enhance antitumor efficacy [110].

### 4.3 Biomaterial Scaffolds for In Situ CAR-T Generation

The development of biomaterial scaffolds for *in situ* CAR-T cell generation constitutes a paradigm shift in cellular immunotherapy, transitioning from systemic intravenous delivery towards localized, tissue-engineered immune cell programming [92]. This strategy harnesses the three-dimensional structural and biochemical properties of engineered biomaterials to construct a defined anatomical niche capable of recruiting, activating, genetically modifying, and expanding endogenous T cells directly within the host [100]. In contrast to systemic administration of viral vectors or nanoparticles, which face challenges of circulatory clearance, off-target distribution, and immune recognition, scaffold-based systems establish a controlled local microenvironment that recapitulates critical features of secondary lymphoid organs, thereby coordinating the multi-stage process of T cell priming, transduction, and differentiation within a confined space [146,147]. Preclinical validation of this concept is robust: customized peptide hydrogels have been engineered to emulate lymphoid microenvironments, enabling rapid CAR-T cell expansion within three days while preserving phenotypic and functional integrity through optimized biophysical and biochemical signaling [111]. Similarly, injectable supramolecular hydrogels facilitate *in situ* T cell programming via sustained release of plasmid CAR DNA and T cell-targeting ligands, effectively reversing immunosuppressive tumor microenvironments and enhancing T cell infiltration in humanized models [112]. Further advancing this paradigm, implantable multifunctional alginate scaffolds (MASTER) have demonstrated the feasibility of full *in vivo* CAR-T cell manufacturing within a single day, generating functional effector cells that control distal tumor growth and exhibit superior persistence compared to conventionally produced counterparts [113]. Collectively, these scaffold-based platforms address key limitations of conventional CAR-T therapy, including protracted *ex vivo* manufacturing, inadequate tumor trafficking, and T cell exhaustion, by establishing localized, bioinstructive niches that streamline production and potentiate anti-tumor immunity.

### 4.4 Physical Priming: Ultrasound and Beyond

Parallel to these biomaterial advances, physical priming modalities, particularly ultrasound-based strategies, are emerging as powerful tools to enhance the precision and efficiency of *in situ* immune cell engineering [101]. These approaches leverage focused energy delivery to defined anatomical regions such as solid tumors or lymphoid tissues, minimizing off-target effects while transiently disrupting biological barriers to facilitate localized delivery of genetic cargo [148,149]. Ultrasound-mediated techniques, prized for their non-invasiveness, deep tissue penetration, and clinical translatability, often employ microbubbles to induce sonoporation, transiently increasing vascular and cellular membrane permeability to enable efficient vector extravasation and transfection [150]. A groundbreaking application of this principle is exemplified by Yoon et al., who developed a mechanogenetic system using focused ultrasound (FUS) to remotely prime solid tumors for CAR-T cell therapy [151]. Their platform incorporates an engineered genetic circuit activated exclusively by the conjunction of FUS-induced mechanical stimulation and subsequent calcium influx in cancer cells, driving localized CD19 expression within a tumor subpopulation. These engineered cells then serve as *in situ* “training centres”, activating systemically delivered CD19-targeting CAR-T cells within the tumor microenvironment and triggering a broad cytotoxic response against the wider malignant population. Validated across *in vitro*, organoid, and *in vivo* models, this platform provides spatiotemporal control over tumor antigen presentation without exogenous co-factors, effectively redirecting CAR-T cell activity against solid tumors and achieving significant suppression of tumor growth. This non-invasive, ultrasound-controlled priming strategy establishes a versatile and safe paradigm for enhancing the specificity and efficacy of cellular immunotherapies, illustrating the transformative potential of integrating physical stimulation with synthetic biology to overcome the barriers to solid tumor treatment.

## 5 Clinical Translation of *In Vivo* CAR-T against Tumors and Autoimmune Diseases

The clinical development of *in vivo* CAR-T therapy is progressing along two parallel paths: one in hematological malignancies, where it builds upon the established success of *ex vivo* CAR-T, and another in solid tumors, a domain where CAR-T therapy has historically faced significant challenges [152]. The clinical landscape is rapidly expanding, with global *in vivo* CAR-T pipelines projected to exceed 100 programs by the end of 2025 [23]. Current clinical trial progress of *in vivo* CAR-T cell therapy for the treatment of tumors is summarized in Table 3.

**Table 3:** Clinical trial progress of *in vivo* CAR-T cells for the treatment of hematological malignancies and solid tumors via clinicaltrial.gov.

Drug Name	Target CARs	Platform	Indication	Phase	Primary Outcomes	Company	Status	NCT Number
JY231	CD19	Anti-CD3 decorated lentivirus	R/R B-cell malignancies	IIT	MTD, incidence of AE after infusion	Shenzen Genocury Ltd.	Recruiting	NCT07065279
INT2104	CD20	Anti-CD7 scFv-decorated lentivirus	R/R B-cell malignancies	I	AE after infusion	Interius BioTherapeutics	Recruiting	NCT06539338
UB-VV111	CD19	Multi-domain anti-CD3, CD80, CD58 decorate lentivirus	R/R B-cell malignancies	I	AE after infusion	Umoja Biopharma	Recruiting	NCT06528301
UB-VV400	CD22	Multi-domain anti-CD3, CD80, CD58 decorate lentivirus	B-cell lymphoma	IIT	MTD	Umoja Biopharma	Recruiting	NCT06743503
ESO-T01	BCMA	Targeted lentiviral particles	R/R Multiple Myeloma	I	DLTs, CRS, TRAE	EsoBiotec	Recruiting	NCT06791681 NCT06691685
OriV508	CD19/BCMA	Lentivirus	R/R hematological malignancies	I	DLTs, CRS, TRAEs	Oricell Therapeutics	Recruiting	NCT07101705
LVIVO-TaVec100	CD19/CD20	Not disclosed	R/R B-cell Malignancies	I	Incidence, severity and type of TEAEs, RP2D	Nanjing Legend Biotech	Recruiting	NCT07002112
JCXH-213	B cell-targeted CAR	tLCNP	R/R B-cell non-Hodgkin lymphoma	IIT	DLT	Immorna	Recruiting	NCT06618313

Note: Abb: CARs: Chimeric Antigen Receptors; R/R: Relapsed/Refractory; scFv: single-chain variable fragment; IIT: Investigator-Initiated Trial; MTD: Maximum Tolerated Dose; AE: Adverse Event; I: Phase I (Clinical Trial); BCMA: B-cell Maturation Antigen; DLTs: Dose-Limiting Toxicities; CRS: Cytokine Release Syndrome; TRAE: Treatment-Related Adverse Events; TEAEs: Treatment-Emergent Adverse Events; RP2D: Recommended Phase 2 Dose; tLCNP: targeted Lipid Nanoparticle.

The global *in vivo* CAR-T sector has entered a phase of accelerated consolidation and clinical validation. Mergers and acquisitions activity vividly reflects growing industry confidence, exemplified by AstraZeneca’s acquisition of EsoBiotec for up to $1 billion (17 March 2025), AbbVie’s combined investment of $3.54 billion in Capstan Therapeutics ($2.1 billion, 30 June 2025) and Umoja Biopharma ($1.44 billion, 01 April 2024), and Gilead/Kite’s purchase of Interius BioTherapeutics ($350 million, 21 August 2025), transactions primarily motivated by the pursuit of proprietary delivery platforms such as ENaBL, targeted lipid nanoparticles (tLNP), and VivoVec™ [23]. In the domain of hematological malignancies, near-term milestones include anticipated Phase II readouts for ESO-T01 in relapsed/refractory multiple myeloma (may in 2026) and for INT2104 in B-cell lymphomas (similarly may in 2026), outcomes that will critically inform assessments of commercial viability. A central strategic debate continues to juxtapose mRNA-LNP and viral vector delivery systems: while viral platforms offer the potential for sustained CAR expression and long-term remission, they are accompanied by genomic integration risks and toxicity profiles that are difficult to modulate; in contrast, mRNA-based systems provide enhanced controllability and a favorable safety profile, though their long-term durability remains unproven. For solid tumors, the period of 2026–2027 may be expected to yield the first Phase I clinical data for multi-targeted mRNA-LNP candidates and locally delivered viral vectors, results that will help delineate feasible translational pathways.

### 5.1 Hematological Malignancies

In the domain of hematological malignancies, the most compelling clinical evidence to date originates from a landmark study conducted by researchers at Wuhan Union Hospital in China. This work documented the world’s first-in-human success of an *in vivo* CAR-T therapy, designated ESO-T01 (BCMA targeted lentiviral particles), in patients with relapsed/refractory multiple myeloma [153]. Targeting BCMA, the therapy yielded objective responses in all four treated patients in this early-phase cohort, with two achieving a stringent complete response. From a safety perspective, a paramount consideration for any novel therapeutic platform, the treatment proved manageable, with no grade 4 or higher non-hematologic toxicities observed, thereby indicating a potentially favorable safety profile at this early stage of development. Remarkably, this regimen mediated the clearance of myeloma cells even in historically challenging compartments such as extramedullary lesions and the cerebrospinal fluid. A particularly significant advantage over conventional *ex vivo* CAR-T products was the elimination of the need for lymphodepleting chemotherapy prior to infusion [153]. While these early results are encouraging, they derive from a small patient group with limited follow-up; confirmation in larger cohorts and with longer observation is necessary to fully establish the efficacy and safety profile of this approach. The considerable promise of this approach has subsequently stimulated substantial corporate investment, exemplified by AstraZeneca’s acquisition of EsoBiotec for up to $1 billion in 17/03/2025, thereby securing access to the BCMA-directed *in vivo* CAR-T candidate ESO-T01, which remains under clinical investigation with preliminary data anticipated by late 2025.

Another notable candidate in this space is INT2104, developed by Interius BioTherapeutics—a company acquired by Gilead’s Kite for $350 million in 21/08/2025, which is engineered to target CD20 and capable of generating both CAR-T and CAR-natural killer (NK) cells *in vivo*. As the first *in vivo* CAR-T candidate to enter human trials (NCT06539338), INT2104 achieved its first patient dosing in Australia in October 2024 and subsequently expanded into European clinical sites in 2025. Designed as an off-the-shelf, single-infusion therapeutic, it aims to treat B-cell malignancies without the need for specialized manufacturing infrastructure, with Kite planning to submit a biologics license application (BLA) in 2026. In the context of B-cell lymphomas and chronic lymphocytic leukemia (CLL), Umoja Biopharma’s candidate UB-VV111, advanced under a $1.44 billion collaboration with AbbVie, received FDA investigational new drug (IND) clearance in July 2024. This candidate employs an engineered viral envelope to deliver a CD19-targeted CAR along with a rapamycin-activated cytokine receptor. A Phase I trial (NCT06528301) is currently ongoing to assess its safety and antitumor activity in relapsed/refractory large B-cell lymphoma (R/R LBCL) and CLL.

The industry landscape for hematological malignancies is characterized by two predominant technological pathways and intensifying global competition. Viral vector-based platforms, such as the ENaBL system utilized by EsoBiotec, emphasize durable efficacy through stable genomic integration and sustained CAR expression, thereby aiming to minimize relapse risks via persistent immune surveillance. In contrast, mRNA-LNP platforms, including Capstan Therapeutics’ CellSeeker™ technology, acquired by AbbVie for $2.1 billion in 06/30/2025, offer superior flexibility, enabling rapid re-dosing upon relapse or facile target switching (e.g., from CD19 to CD22) to overcome antigen escape. Capstan’s lead candidate, CPTX2309, which employs a CD8-targeted tLNP to deliver mRNA encoding a CD19-directed CAR, entered Phase I trials in June 2025, initially for autoimmune diseases but with clear translational potential in B-cell malignancies. Domestically, Chinese biopharmaceutical companies are accelerating their development efforts: MagicRNA published clinical findings in The New England Journal of Medicine (2025) that validated an mRNA-LNP-based *in vivo* CAR-T approach for refractory systemic lupus erythematosus [154], while Immorna is investigating its candidate JCXH-213 under outpatient-administered maintenance therapy paradigms. Globally, the *in vivo* CAR-T pipeline targeting hematologic cancers now exceeds 20 assets, with approximately 60% in early-stage clinical or preclinical development, and CD19 and BCMA representing the most frequently targeted antigens.

### 5.2 Solid Tumors

Solid tumors present significantly greater challenges for *in vivo* CAR-T therapy, primarily due to tumor heterogeneity, the immunosuppressive TME, and the risks of on-target/off-tumor toxicity. However, the use of LNP platforms introduces distinct pharmacodynamic properties that directly influence the safety profile and dosing strategy. Due to the transient nature of mRNA-encoded CAR expression, LNP-based therapies may limit the duration of immune activation, thereby potentially reducing the risk of prolonged cytokine release syndrome or persistent on-target toxicity. This transient expression also allows for more flexible and titratable dosing schedules, including the possibility of repeated administrations to sustain anti-tumor activity or address antigen escape, a feature not as readily achievable with integrating viral vectors [155]. Nevertheless, it is important to note that this transient expression can also be a critical drawback in the context of solid tumors, where sustained persistence is often required for therapeutic efficacy. Despite these barriers, early clinical exploration and preclinical breakthroughs are laying the groundwork for potential future effectiveness [90].

Hefei RNAlfa Biological Technology Co., Ltd. (RNAlfa) has initiated an exploratory clinical study of its AFN4801, first logic-gated *in vivo* CAR-T therapy for advanced solid tumors. This represents the world’s first clinical trial for an *in vivo* CAR-T therapy incorporating a dual-antibody “AND-gate” control system. Utilizing its proprietary mRNA delivery platform, the therapy aims to directly program a patient’s T cells inside the body *in vivo*, bypassing the complex and costly traditional process of cell extraction, external modification, and reinfusion *ex vivo* using previous established low immunogenicity LNP system, which can induce the functional reprogramming of immune cells *in vivo* [156].

This innovative approach has the potential to transform CAR-T treatment from a personalized, hospital-based procedure into a more accessible, off-the-shelf, and potentially outpatient-administered therapy. Preclinical data suggests reductions in both manufacturing time (from weeks to potentially same-day use) and projected cost per dose. If validated clinically, this could dramatically improve the accessibility of CAR-T treatment. It is important to note that this is an early-stage exploratory study, and its ultimate clinical efficacy and safety profile require further validation.

### 5.3 Autoimmune Diseases

*In vivo* CAR T-cell therapy has emerged as a transformative strategy for autoimmune diseases, fundamentally circumventing a pivotal limitation of conventional *ex vivo* CAR-T products [142]: the mandatory use of toxic lymphodepleting conditioning chemotherapy [157]. This prerequisite regimen, employed to suppress the host immune system and enable engraftment of externally manufactured cells, carries substantial risks of infection, organ toxicity, and prolonged cytopenias, a particularly critical concern in the treatment of non-malignant conditions such as autoimmunity [158,159]. By utilizing targeted delivery vectors, including antibody-conjugated LNPs or engineered lentiviral particles, the *in vivo* approach directly reprograms a patient’s endogenous T cells to express CARs *in situ*. This paradigm not only obviates the need for preconditioning chemotherapy but also transforms the therapeutic workflow from a protracted, multi-week cellular manufacturing process into a more streamlined, “off-the-shelf” pharmacologic administration, thereby enhancing both safety and potential scalability. The most compelling clinical validation of this approach was recently reported: a first-in-human study of HN2301, an LNP-based *in vivo* anti-CD19 CAR-T therapy, in five patients with refractory systemic lupus erythematosus (SLE). A single low-dose (2 mg) intravenous infusion, administered without prior lymphodepletion, led to detectable peripheral CAR-T cell levels (10%) within six hours in the first patient, accompanied by a rapid and profound reduction in B cells. The treatment was well-tolerated, with no severe cytokine release syndrome or neurotoxicity observed, and was associated with reduced disease activity [154]. In parallel, the field is rapidly expanding, as exemplified by the *in vivo* CAR-T candidate JY231. This therapy employs a lentiviral vector to generate CD19-targeting CAR-T cells *in situ* and is designed to forego traditional lymphodepleting chemotherapy. JY231 is currently under investigation in two planned early-phase clinical trials (NCT06243159, NCT06887985) for a spectrum of refractory autoimmune conditions, including SLE, Sjögren’s syndrome, and systemic sclerosis. Collectively, these advances underscore the potential of *in vivo* CAR-T platforms to deliver precise, potent immune modulation with an improved safety and practicality profile, paving the way for broader clinical application in autoimmunity.

## 6 Challenges and Future Directions

### 6.1 Unresolved Biological Barriers

Substantial biological barriers continue to impede progress, especially in solid tumors [160]. The immunosuppressive tumor microenvironment constitutes a particularly complex challenge that *in vivo* CAR approaches must address [1]. In contrast to hematologic malignancies, where target cells circulate freely or reside in lymphoid tissues, solid masses establish both physical and chemical obstacles that restrict CAR cell infiltration and functionality. An aberrant vascular network and compact extracellular matrix collectively hinder the efficient trafficking of systemically delivered vectors and the subsequent migration of effector cells generated *in vivo* [43]. Moreover, even when CAR-T cells successfully penetrate the tumor stroma, they encounter a hostile niche saturated with immunosuppressive cytokines, metabolic rivals, and regulatory immune populations that synergistically drive T cell dysfunction and exhaustion [161]. Beside the *in vivo* CAR-T, *in vivo* reprogramming of myeloid cells, a strategy under investigation by entities such as Myeloid Therapeutics and the Carisma/Moderna collaboration, offers a viable avenue for remodeling the tumor microenvironment from the inside, capitalizing on the innate ability of macrophages to infiltrate hypoxic tumor regions. However, the long-term efficacy and overall therapeutic impact of this tactic await confirmation through rigorous clinical studies. Beyond the immunosuppressive landscape, antigen escape remains a dominant mechanism of therapeutic resistance [49]. The inherently transient expression of mRNA-encoded CARs, though beneficial from a safety standpoint, may require carefully orchestrated redosing schedules or simultaneous targeting of multiple tumor-associated antigens to prevent the expansion of antigen-negative malignant clones. For viral vector-based systems that enable sustained CAR expression, the central challenge involves mounting a sufficiently broad and potent initial antitumor response to eliminate heterogeneous cancer cell populations before clonal selection can take place [162].

### 6.2 Safety and Regulatory Considerations

In parallel, the safety and regulatory landscape for these innovative therapeutic agents is being actively defined. The integration of a highly potent CAR payload with advanced delivery technologies creates a distinctive and not yet fully understood risk profile, calling for a preemptive and sophisticated regulatory science framework [163]. With lentiviral-based platforms, primary concerns relate to the potential for uncontrolled expansion and prolonged persistence of genetically altered T cells. The integrating characteristic of the viral payload warrants serious, albeit low-probability, considerations of genotoxicity and insertional mutagenesis, justifying long-term patient monitoring as recommended in recent FDA guidance documents [164]. Rare instances of T-cell lymphomas following conventional *ex vivo* CAR-T therapy highlight the importance of thorough vector integration site analysis and clonal tracking in clinical trials involving *in vivo* viral CAR products [77]. Conversely, the safety characteristics of LNP-RNA systems are governed by the transient pharmacokinetics of the CAR construct and the inherent reactogenicity of the nanoparticle formulation. Risks such as acute infusion reactions, complement activation-related pseudoallergy, and hepatotoxicity stemming from the natural liver tropism of early-generation LNPs require diligent clinical management [165]. Mitigation strategies center on lipid redesign, incorporating ionizable lipids with improved biodegradability and reduced inflammatory potential (e.g., SM-102, ALC-0315 used in mRNA vaccines), and surface functionalization with polyethylene glycol (PEG) or other hydrophilic polymers to decrease protein opsonization and macrophage uptake [90,166]. A pivotal unresolved issue is whether the limited duration of CAR expression with mRNA platforms will adequately prevent severe inflammatory adverse events, such as cytokine release syndrome and immune effector cell-associated neurotoxicity syndrome, or simply modify their temporal onset and duration [167]. Emerging accounts of localized immune effector cell-associated toxicity syndrome in autoimmune patients receiving CAR-T cells further suggest the possibility of novel, tissue-specific toxicities that could also emerge with *in vivo* delivery systems. Regulatory bodies therefore face the challenge of adapting existing cell and gene therapy frameworks to accommodate these hybrid products, striking a balance between comprehensive safety evaluation and the flexibility needed to foster innovation [168]. This effort will likely entail the creation of novel biomarkers for early toxicity detection, the adoption of risk-adjusted monitoring approaches, and possibly the integration of built-in safety mechanisms such as ligand-activated kill switches or microRNA-regulated CAR expression to achieve precise spatiotemporal control over therapeutic activity [169].

To translate conceptual safety frameworks into actionable clinical development, platform-specific trial design recommendations are essential. For integrating viral vectors (e.g., lentivirus, certain engineered AAVs), long-term monitoring must include systematic integration site analysis performed at key timepoints, such as peak expansion, 6 months, and annually thereafter, for at least 15 years to screen for clonal expansion suggestive of insertional risk [98]. A tiered safety schedule is recommended: frequent pharmacokinetic/pharmacodynamic and toxicity assessments (weekly to bi-weekly) during the initial 2–3 months, followed by quarterly reviews in the first year and annual evaluations thereafter [20]. For transient platforms like mRNA-LNPs, the focus shifts to acute reactogenicity and cytokine-driven events; protocols should mandate real-time biomarker tracking (e.g., ferritin) coupled with predefined management algorithms for CRS and ICANS, aligned with the transient pharmacokinetics of CAR expression. A critical consideration for viral vectors, especially AAV, is pre-existing anti-capsid immunity; baseline neutralizing antibody testing should be mandatory, with trial designs incorporating exclusion thresholds, stratification, or dose-adjustment strategies. Across platforms, early-phase trials should employ adaptive designs, including safety review committees with predefined stopping rules and pharmacokinetic-guided dose escalation, to efficiently characterize the novel risk-benefit profile of *in vivo* generated CAR-T therapies [81].

### 6.3 Emerging Combination Therapy Strategies

In light of these biological and safety impediments, research emphasis is increasingly shifting toward combination therapy strategies. The future clinical value of *in vivo* CAR interventions will probably depend on their integration into rationally constructed combination regimens rather than their use as monotherapies [8]. The inherent modularity of the platform, especially the LNP-RNA format, is exceptionally well-suited for such combinatorial applications. Co-encapsulation or concurrent administration of mRNAs encoding immunostimulatory molecules, for example, constitutively active cytokine receptors like IL-7 or IL-15, dominant-negative TGF-β receptors, or bispecific T-cell engager, can be employed to fortify CAR cells against the suppressive tumor milieu and augment their antitumor effectiveness [170]. Another emerging frontier involves the coordinated *in vivo* engineering of multiple immune cell lineages; one potential approach is the simultaneous generation of CAR-T cells for direct tumor elimination and CAR-myeloid cells for microenvironment remodeling and antigen presentation. Pairing *in vivo* CAR therapy with established immunotherapies, especially immune checkpoint inhibitors, represents a rational and encouraging direction [171]. Preclinical findings from Carisma Therapeutics indicate that CAR-macrophages can enhance the sensitivity of solid tumors to anti-PD-1 treatment, and analogous synergistic effects are expected with *in vivo*-generated CAR-T cells. Furthermore, combining *in vivo* CAR therapy with conventional treatment modalities such as radiotherapy or targeted agents could establish favorable immunogenic conditions that disrupt localized immune tolerance and trigger a systemic antitumor response [172]. The fullest realization of this combinatorial potential may emerge from the creation of “multi-input” genetic circuits, in which CAR expression or activation is controlled by logic gates requiring the detection of multiple tumor-specific antigens, thereby attaining an exceptional degree of tumor selectivity and safety.

### 6.4 Scalability and Manufacturing of Viral and Non-Viral Platforms

The scalable manufacture of *in vivo* CAR-T therapies poses distinct challenges for viral and non-viral platforms. Production of lentiviral and AAV vectors remains resource-intensive, relying on complex cell-culture systems, stringent purification, and rigorous testing for replication-competent viruses, factors that limit yield and increase cost. In contrast, LNP production benefits from processes established for mRNA vaccines; however, achieving consistent T-cell specific targeting under GMP conditions, via precise antibody conjugation or optimized ionizable lipids, introduces additional complexity [173]. Practical scale-up must address key cost drivers, including viral vector titers, LNP encapsulation efficiency, and the need for specialized cold-chain logistics, especially for mRNA-based formulations [174]. Batch-to-batch variability in vector potency or nanoparticle properties remains a critical quality control hurdle, with potential implications for clinical reproducibility [175]. Although LNPs may offer a more modular and potentially lower-cost manufacturing route, global access continues to be constrained by high infrastructure requirements, uneven distribution of GMP capacity, and the substantial expense of advanced therapy production, factors that risk perpetuating disparities between high and low resource settings [155,176]. Looking ahead, realistic development timelines must balance promising early-phase results with the stringent demands of pivotal trials and regulatory review. While first-in-human successes have been reported and several candidates are progressing through Phase I/II studies, broader clinical adoption, particularly in solid tumors, will likely necessitate iterative delivery optimization, demonstration of durable efficacy, and comprehensive safety monitoring.

## Data Availability

Not applicable.

## Abbreviations

**Terms**: **Full** **Name**
CAR-T: Chimeric Antigen Receptor T-Cell
BCMA: B Cell Maturation Antigen
scFv: Single-Chain Variable Fragment
MHC: Major Histocompatibility Complex
TME: Tumor Microenvironment
ECM: Extracellular Matrix
Tregs: Regulatory T Cells
MDSCs: Myeloid-Derived Suppressor Cells
CRS: Cytokine Release Syndrome
ICANS: Immune Effector Cell-Associated Neurotoxicity Syndrome
IEC-HS: Immune Effector Cell-Associated Hemophagocytic Syndrome
GMP: Good Manufacturing Practice
LNPs: Lipid Nanoparticles
AAV: Adeno-Associated Virus
VSV-G: Vesicular Stomatitis Virus Glycoprotein
MV: Measles Virus
NiV: Nipah Virus
DARPin: Designed Ankyrin Repeat Protein
SINV: Sindbis Virus
NHPs: Non-Human Primates
ACG: AAV-Delivered CAR Gene Therapy
PEI: Polyethyleneimine
PDMAEMA: Poly(2-dimethylaminoethyl methacrylate)
PβAE: Poly(β-amino ester)
tDC-Exo: Tumor Antigen-Stimulated Dendritic Cell-Derived Exosomes
MASTER: Multifunctional Alginate Scaffolds for T-Cell Engineering and Release
FUS: Focused Ultrasound
R/R: Relapsed/Refractory
LBCL: Large B-Cell Lymphoma
CLL: Chronic Lymphocytic Leukemia
IND: Investigational New Drug
BLA: Biologics License Application
tLNP: Targeted Lipid Nanoparticle
DLTs: Dose-Limiting Toxicities
TRAE: Treatment-Related Adverse Events
RP2D: Recommended Phase 2 Dose
TEAEs: Treatment-Emergent Adverse Events
TROP2: Trophoblast Cell-Surface Antigen 2
GPC3: Glypican-3
HER2: Human Epidermal Growth Factor Receptor 2
TGF-β: Transforming Growth Factor-Beta
IL-6: Interleukin-6
IL-1β: Interleukin-1 Beta
DAMPs: Damage-Associated Molecular Patterns
APCs: Antigen-Presenting Cells
EGFR: Epidermal Growth Factor Receptor
TRAC: T Cell Receptor Alpha Constant
mRNA: Messenger RNA
DNA: Deoxyribonucleic Acid
FDA: Food and Drug Administration
